# Association between a single-pass whole-body computed tomography policy and survival after blunt major trauma: a retrospective cohort study

**DOI:** 10.1186/1757-7241-19-73

**Published:** 2011-12-09

**Authors:** Martin Hutter, Alexander Woltmann, Christian Hierholzer, Christian Gärtner, Volker Bühren, Dirk Stengel

**Affiliations:** 1 Department of Trauma and Orthopedic Surgery, Berufsgenossenschaftliche Unfallklinik Murnau, Prof.-Küntscher-Str. 8, 82418 Murnau, Germany; 2 Department of Radiology, Berufsgenossenschaftliche Unfallklinik Murnau, Prof.-Küntscher-Str. 8, 82418 Murnau, Germany; 3 Center for Clinical Research, Department of Trauma and Orthopedic Surgery, Unfallkrankenhaus Berlin, Warener Str. 7, 12683 Berlin, Germany

## Abstract

**Introduction:**

Single-pass, whole-body computed tomography (pan-scan) remains a controversial intervention in the early assessment of patients with major trauma. We hypothesized that a liberal pan-scan policy is mainly an indicator of enhanced process quality of emergency care that may lead to improved survival regardless of the actual use of the method.

**Methods:**

This retrospective cohort study included consecutive patients with blunt trauma referred to a trauma center prior to (2000 to 2002) and after (2002 to 2007) the introduction of a liberal single-pass pan-scan policy. The overall mortality between the two periods was compared and stratified according to the availability and actual use of the pan-scan. Logistic regression analysis was employed to adjust mortality estimates for demographic and injury-related independent variables.

**Results:**

The study comprised 313 patients during the pre-pan-scan period, 223 patients after the introduction of the pan-scan policy but not undergoing a pan-scan and 608 patients undergoing a pan-scan. The overall mortality was 23.3, 14.8 and 7.9% (*P *< 0.001), respectively. By univariable logistic regression analysis, both the availability (odds ratio (OR) 0.57, 95% confidence interval (CI): 0.36 to 0.90) and the actual use of the pan-scan (OR 0.28, 95% CI: 0.19 to 0.42) were associated with a lower mortality. The final model contained the Injury Severity Score, the Glasgow Coma Scale, age, emergency department time and the use of the pan-scan. 2.7% of the explained variance in mortality was attributable to the use of the pan-scan. This contribution increased to 7.1% in the highest injury severity quartile.

**Conclusions:**

In this study, a liberal pan-scan policy was associated with lower trauma mortality. The causal role of the pan-scan itself must be interpreted in the context of improved structural and process quality, is apparently moderate and needs further investigation with regard to the diagnostic yield and changes in management decisions. (The Pan-Scan for Trauma Resuscitation [PATRES] Study Group, ISRCTN35424832 and ISRCTN41462125)

## Introduction

With the recent proclamation of the Decade of Action for Road Safety, the United Nations and the World Health Organization stressed the importance of severe trauma, its prevention and effective treatment as a global public health concern [1-3].

The promising trend towards a lower than expected mortality rate in the industrial countries over the past years should not conceal that a plateau of a 12% mortality has been reached in patients with multiple trauma [4]. Of even more concern is the diversity in outcomes across different centers, with standardized mortality ratios ranging from 0.6 to 1.1 [5].

The goal of primary trauma work-up is apparently simple: injuries that are ultimately life-threatening must be identified with high diagnostic accuracy. Significant injuries missed during the primary survey have been named the "nemesis of the trauma surgeon" [6] and may be the deciding factor for survival or death [7-11]. There is still a high degree of variability in the reported incidence of missed injuries ranging from 1 up to 39% [12], stressing the need for continuing improvement of trauma algorithms.

Staged diagnostic work-up, including physical examination, focused abdominal sonography for trauma (FAST), and plain X-rays of the chest, pelvis, and vertebral column, followed by selective computed tomography (CT) of the cranium and suspected body regions, remains the accepted standard of care in most trauma centers and is, in accordance with the principles of Advanced Trauma Life Support (ATLS^®^) [13], the most frequently applied and highly standardized trauma algorithm worldwide.

However, clinical findings like belt marks are unreliable [14], and plain radiographs and FAST have low sensitivity in ruling out organ injuries [15-21].

Single-pass, contrast-enhanced, whole-body computed tomography, coined the pan-scan, has emerged as a new way of depicting injury patterns in patients with multiple trauma [22-29].

In Europe, many emergency departments have now been converted to allow for shorter distances between the trauma bay and the CT scanner. Propagators of the primary pan-scan stress the rapidity and precision with which conclusive information for management decisions can be obtained.

Opponents argue that transfer and repositioning puts patients at risk of hemodynamic deterioration. More importantly, an estimated 0.4% of all cancers in the United States may be attributable to the radiation from CT scans, and given the increasing frequency of scans this estimate might now range between 1.5 and 2.0% [30]. Since mostly patients under 40 years of age are affected by multiple trauma, trauma teams in particular are responsible for balancing the potential benefits against the possible harms of the pan-scan, and for minimizing the number of unnecessary scans.

Although there is evidence that the pan-scan increases the detection rate of otherwise non-apparent injuries and that it shortens the transfer-interval between operating theatre and intensive-care unit (ICU) [31], its effectiveness with regard to survival remains unproven. In a recent analysis of the German Trauma Registry, the increasing use of the pan-scan was causally linked to an improved ratio of observed to expected deaths [32]. The results of this study were called into question because of the susceptibility of the findings to stage-migration, the observational nature of the registry and unclear rates of misclassification [33].

The primary objective of the current study was to determine the causal contribution of pan-scan imaging to overall survival after the implementation of a new algorithm that allowed trauma teams to schedule patients to a pan-scan 24/7 according to their discretion.

## Materials and methods

This was a retrospective cohort study which compared survival after severe blunt trauma before and after the introduction of a liberal pan-scan-policy at a dedicated maximum-care, high-volume trauma center in the most southern part of Germany which is owned and run by the Federal Employer's Liability Insurance Association ("Berufsgenossenschaft"). The study was approved by the institutional review board, and a consent waiver was granted because of the use of routinely collected hospital data.

This work was part of the multifaceted, multi-center PATRES (Pan-Scan for Trauma Resuscitation, ISRCTN35424832 and ISRCTN41462125) project that investigates the different levels of diagnostic test research pertaining to the whole-body scan in early trauma care. Briefly, one aspect of the project is to determine the diagnostic accuracy of the method by correlating the initial scans to a synopsis of clinical follow-up, subsequent imaging and surgical notes. Another integral part of the project is a comparison between the surgeon's pre-test probability of injuries prior to CT and later pan-scan findings. Finally, two independent cohort studies have been conducted at two trauma centers in the most northern and most southern regions of Germany. Data from one of these studies form the basis of this report.

### Control (non pan-scan) period and cohort

Until April 2002, the initial diagnostic evaluation of patients with multiple trauma followed ATLS^® ^principles and included a structured physical examination, a FAST scan, plain X-rays of the chest and pelvis in the anterior-posterior view, and anterior-posterior and lateral radiographs of the entire vertebral column, always followed by cranial CT. FAST was performed by board-certified trauma surgeons. Additional x-ray or CT scans of selected body regions were available if requested at the discretion of the trauma leader. Data were recorded on standardized TraumaRegister^D ^case report forms issued by the German Society of Trauma Surgery (Deutsche Gesellschaft für Unfallchirurgie e.V.) [5]. After completion of the trauma survey at the emergency department, patients were transferred to the operating theatre or the ICU.

### Intervention (pan-scan) period and cohort

#### Triage criteria

In May 2002, the emergency department of the institution was redesigned, and a four-row multi-detector CT scanner (LightSpeed plus^®^, General Electric, Neu-Isenburg, Germany) was installed close to the trauma bay. In April, 2006 the device was replaced by a 64-row multi-detector CT scanner (General Electric^®^, Lightspeed VCT, Neu-Isenburg, Germany).

Trauma surgeons were encouraged to schedule their patients for a pan-scan as the primary imaging tool after resuscitation, a brief physical check-up and FAST examination. All patients who had been exposed to a blunt high-velocity incident with a high pre-test probability of major trauma based on the mechanism of injury were eligible for a pan-scan. This included subjects involved in vehicle crashes with death of other occupants, extrication, or need for technical rescue, falls from a height, pedestrians, bicyclists, or bikers hit by larger vehicles, as well as subjects with unknown injury mechanisms and those with abnormal vital parameters. Patients scheduled for the pan-scan had to be hemodynamically stable after resuscitation with a systolic blood pressure of at least 90 mmHg.

Pregnant women were excluded from undergoing a pan-scan. Super-obese patients with a body weight of > 200 kg were excluded as well, given the upper load bearing capacity of the CT table.

#### Pan-scan imaging protocol

The following procedures and scanning parameters applied to the trauma pan-scan: tube voltage 120 kV, native scan of the head and the cervical spine with the upper extremities positioned adjacent to the body at axial slice thickness of 0.62 mm, followed by multi-planar reconstruction with a slice thickness of 2 mm. After repositioning the arms over the head, 100 ml of iopromide (Ultravist^® ^300, Bayer-Schering, Germany, containing 300 mg/ml of iodine) were injected at a flow rate of 4 ml/s, and the diagnostic procedure was completed by contrast-enhanced imaging in the arterial phase of the thorax, abdomen, pelvis, vertebral column and the upper part of the legs. Additionally, a venous phase of the intra-abdominal organs was performed. The slice thickness was 0.62 mm for acquisition of raw data, 2.5 mm for axial, 3.0 mm for coronal, and 5.0 mm for sagittal reconstruction of the trunk. For the vertebral column, a slice thickness of 2.0 mm was employed in either plane to guarantee high resolution in this sensible area.

All patients were closely monitored during the scanning procedure, and the trauma team had full access to patients in case of signs of hemodynamic instability. In case of safety concerns or the need for life-saving interventions (e.g. tube thoracostomy for tension pneumothorax) the pan-scan was immediately stopped.

### Data recording

To determine the impact of the new protocol on outcomes, two cohorts of consecutive patients admitted to the hospital before (between January 2000 and April 2002) and after the introduction of the pan-scan algorithm (between May 2002 and December 2007) were compared. Eligible subjects were identified from the local trauma database which, in addition to external quality assurance by the federal TraumaRegister^D^, contained demographic and injury-related items, physiological data on admission and variables describing the clinical progress until discharge.

### Statistical analysis

Data are presented as means, medians, proportions, risks and ratios with appropriate measures of variability according to the underlying data quality and distribution (e.g. ranges, interquartile ranges, and standard deviations). 95% confidence intervals (CI) were calculated whenever it was methodologically sound. Baseline imbalances between groups were assessed by analysis of variance for continuous measures and the χ^2 ^test for categorical variables.

The contribution of individual variables to overall mortality was modeled by univariable and multivariable logistic regression analysis. Candidate predictors were included using a screening *P *value of 0.2, and excluded at a *P *value of 0.1. Model fit was evaluated by the Hosmer-Lemeshow test for goodness of fit. The variance in mortality explained by the best set of variables was assessed by areas under the receiver operating characteristics curve (ROC).

In addition, separate models were computed for subsets of patients with increasing trauma load, as categorized by ISS quartiles. STATA 10.0 statistical software was employed for all analyses.

## Results

Between 1 January 2000 and 31 December 2007, 1912 patients were admitted to the study site, 1144 (59.8%) of which were immediately transferred from the scene of accident.

313 patients were admitted during the control period of staged work-up (January 2000 to April 2002), and 831 patients were admitted during the intervention period of liberal pan-scan use (May 2002 to December 2007). It was noteworthy that 223 (26.8%) admitted during the latter period did not undergo a pan-scan despite its availability at the discretion of the surgeon on call. Thus, the study sample comprised patients who did not undergo a pan-scan due to the unavailability of the method (n = 313), patients who were eligible but not scheduled for the pan-scan (n = 223) and eligible patients who underwent a pan-scan (n = 608).

There were 19 patients (2.3%) in the intervention period who underwent a subsequent pan-scan after early termination of the resuscitation protocol. Eight patients were scheduled for emergency laparotomy due to a massive hemoperitoneum detected by FAST sonography prior to the pan-scan, and with another eleven patients there were either trepanation and evacuation of intracranial hematomas or other reasons for stopping the pan-scan early.

Overall, the study sample included 851 men (74.4%) and 293 women (25.6%). The mean age was 44.9 (standard deviation 20.3) years, and the ISS averaged 26.4 (11.7) points (Table 1). Patients undergoing a pan-scan had the highest mean ISS, and were more likely to suffer from leading injuries (AIS ≥ 4) to the trunk, especially the chest and abdomen.

**Table 1:** Characteristics of all patients, by time period.

	Control Cohort (2000 to 2002)	Intervention Cohort (2002 to 2007)		*P *value¶
		Pan-scan not performed	Pan-scan performed	
	n = 313	n = 223	n = 608	
Mean age, years*	43.5 (20.7)	49.6 (21.9)	43.9 (19.3)	< 0.001
Sex†				0.815
Male	234 (75)	169 (76)	448 (74)	
Female	79 (25)	54 (24)	160 (26)	
Mean GCS*	10.5 (4.9)	12.3 (4.1)	11.2 (4.6)	< 0.001
Mean heart rate, bpm	86. 7 (19.9)	85.2 (18.9)	84.6 (17.9)	0.327
Mean systolic pressure, mmHg	126.4 (26.7)	130.5 (28.3)	120.1 (24.9)	< 0.001
Mean hemoglobin, mmol/l*	7.2 (1.9)	8.2 (1.7)	7.5 (1.7)	< 0.001
Mean thrombocyte count × 10^9 ^/l*	189 (81)	204 (103)	191 (66)	0.065
Mean prothrombin time, per cent*	71.3 (27.1)	82.1 (23.9)	74.8 (20.3)	< 0.001
Mean no. of PRB transfusions*	9.3 (10.9)	7.9 (9.2)	7.1 (6.6)	0.134
Mean ISS*	26.4 (12.2)	21.3 (8.5)	28.3 (11.8)	< 0.001
ISS quartiles†				< 0.001
First, 0 to 18	88 (28)	105 (47)	132 (22)	
Second, 19 to 25	111 (35)	89 (40)	187 (31)	
Third, 26 to 33	36 (12)	13 (6)	110 (18)	
Fourth, 34 to 75	78 (25)	16 (7)	179 (29)	
ISS ≥ 16†	282 (90)	216 (97)	578 (95)	0.001
ED time, min				
Mean*	144.7 (115.8)	95.7 (63.1)	83.5 (49.2)	< 0.001
Median‡	106 (60 - 213)	80 (48 - 133)	74 (52 - 103)	< 0.001
Head injuries†	210 (67)	143 (64)	330 (54)	< 0.001
Mean AIS_Head_*	3.5 (1.2)	4.1 (0.8)	3.6 (1.1)	< 0.001
Chest injuries†	163 (52)	66 (30)	407 (67)	< 0.001
Mean AIS_Chest_*	3.3 (1.0)	3.5 (0.9)	3.7 (0.8)	< 0.001
Abdominal trauma†	61 (19)	19 (9)	124 (20)	< 0.001
Mean AIS_Abdomen_*	3.4 (1.0)	4.1 (1.1)	3.2 (1.1)	0.004
Spine fractures†	130 (42)	56 (25)	342 (56)	< 0.001
Mean AIS_Spine_*	3.3 (1.2)	3.4 (1.0)	3.2 (1.1)	0.197
Mean length of ICU stay, days*	16.2 (17.0)	9.9 (10.6)	16.2 (17.3)	< 0.001
Mean no. of surgical procedures*	4.1 (2.9)	2.9 (3.5)	4.1 (3.9)	0.001
ARDS†	68 (22)	19 (9)	120 (20)	< 0.001
MOF†	8 (3)	22 (10)	119 (20)	< 0.001
Sepsis†	12 (4)	2 (1)	21 (3)	0.081
Mortality†	73 (23)	33 (15)	48 (8)	< 0.001

Values in parentheses are *standard deviations, †percentages or ‡interquartile ranges.

¶*P *values for continuous variables were computed using analysis of variance, and for binary variables using the χ^2 ^test. For skewed distributions and medians, the Kruskal-Wallis test was employed. AIS, Abbreviated Injury Scale; ARDS, respiratory distress syndrome; ED, emergency department; GCS, Glasgow Coma Scale; ISS, Injury Severity Score; ICU, intensive care unit; MOF, multiple organ failure; PRB, units of packed red blood.

**Figure 1:**
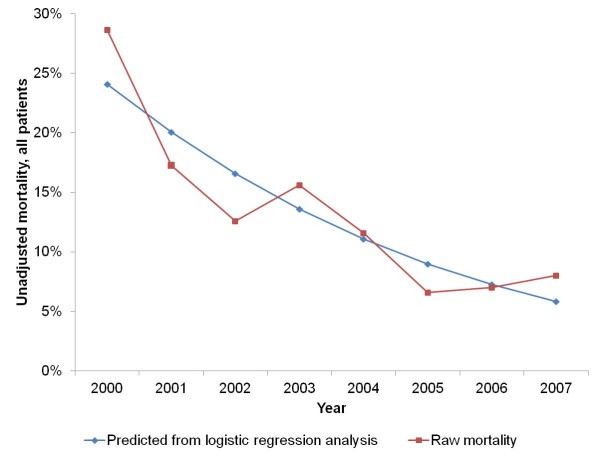
**Time trend in overall mortality**. Unadjusted data.

Raw mortality decreased steadily from the year 2000 (28.7%, 95% CI: 21.4 to 36.8%) to 2007 (8.0%, 95% CI: 4.2 to 13.6%, Figure 1).

With regard to the observational periods, raw mortality decreased from 23.3% (95% CI: 18.8 to 28.4%) in the control period to 9.7% (95% CI: 7.8 to 12.0%) in the intervention period. Of note, there was a marked decline in mortality from the control to the intervention period regardless of the use of the pan-scan (14.8%, 95% CI: 10.4 to 20.1%). This translated to an odds ratio (OR) of 0.57 (95% CI: 0.36 to 0.90, *P *= 0.015).

Likewise, the time patients spent in the emergency department decreased significantly from the intervention to the control period (mean 144.7 vs. 86.8 min; mean difference 57.9 min, 95% CI: 47.6 to 68.2 min; *P *< 0.001). The time reduction by the actual use of the pan-scan during the intervention period was 12.2 min (95% CI: 3.7 to 20.6 min, *P *= 0.005). Similar time trends were observed among all except the fourth ISS quartile in which no difference was observed between patients eligible for and actually undergoing a pan-scan (Table 2).

**Table 2:** Mean duration of emergency department treatment (in min) stratified according to ISS quartiles and periods of observation.

	January 2000 to April 2002	May 2002 to December 2007	
ISS quartile	Pan-scan not available	Pan-scan available but not performed	Pan-scan performed
First, 0 to 18	173.9 (147.1 to 200.8)	96.9 (84.0 to 109.7)	85.1 (77.0 to 93.1)
Second, 19 to 25	145.7 (122.3 to 169.1)	99.1 (85.0 to 113.1)	89.8 (80.8 to 98.7)
Third, 26 to 33	137.4 (95.9 to 178.8)	113.8 (79.3 to 148.4)	83.4 (74.1 to 92.7)
Fourth, 34 to 75	112.8 (87.6 to 138.1)	50.7 (33.3 to 68.2)	76.3 (70.2 to 82.4)

Values in parentheses are 95% CI. In first approximation, a significant difference in the mean duration of emergency department time at the 5% threshold between individual subgroups can be assumed if 95% confidence limits do not overlap.

ISS, Injury Severity Score.

By univariable analysis, both the availability and the actual use of the pan-scan were associated with a decrease in mortality (Table 3). The best-fitted multivariable model comprised the ISS, GCS, age, duration of emergency department care and pan-scan diagnostics as independent predictors of mortality (Table 4). There was no evidence for a lack of goodness of fit (*P *= 0.897). Also, there was no interaction between the duration of emergency department care and the use of the pan-scan.

**Table 3:** Results from univariable logistic regression analysis of mortality.

Variable	Odds Ratio	*P *value
Pan-scan available	0.57 (0.36 to 0.90)	0.015
Pan-scan performed	0.28 (0.19 to 0.42)	< 0.001
Year	0.79 (0.73 to 0.86)	< 0.001
Age, years	1.02 (1.01 to 1.03)	< 0.001
Heart rate, bpm	1.01 (1.00 to 1.02)	0.029
Systolic blood pressure, mmHg	0.99 (0.99 to 1.00)	0.025
GCS	0.81 (0.78 to 0.84)	< 0.001
ISS	1.05 (1.03 to 1.06)	< 0.001
ED time, min	0.99 (0.99 to 1.00)	< 0.001
Mean AIS_Head_	3.29 (2.41 to 4.49)	< 0.001
Mean AIS_Abdomen_	2.84 (1.83 to 4.41)	< 0.001
Mean AIS_Spine_	1.40 (1.06 to 1.87)	0.019
Hemoglobin, mmol/l	0.72 (0.66 to 0.79)	< 0.001
Thrombocyte count × 10^9 ^/l	1.00 (1.00 to 1.00)	0.003
Prothrombin time, %	0.97 (0.97 to 0.98)	< 0.001
No. of PRB transfusions	1.06 (1.03 to 1.10)	< 0.001

Values in parentheses are 95% CI. AIS, Abbreviated Injury Scale; ED, emergency department; GCS, Glasgow Coma Scale; ISS, Injury Severity Score; PRB, units of packed red blood.

**Table 4:** Results from multivariable logistic regression analysis of mortality.

Variable	Odds Ratio	*P *value
Pan-CT available	0.65 (0.36 to 1.19)	0.164
Pan-CT performed	0.17 (0.10 to 0.28)	< 0.001
Age, years	1.04 (1.03 to 1.05)	< 0.001
GCS	0.81 (0.78 to 0.85)	< 0.001
ISS	1.04 (1.02 to 1.06)	< 0.001
ED time, min	0.99 (0.99 to 1.00)	< 0.001

Values in parentheses are 95% CI. ED, emergency department; GCS, Glasgow Coma Scale; ISS, Injury Severity Score.

**Figure 2:**
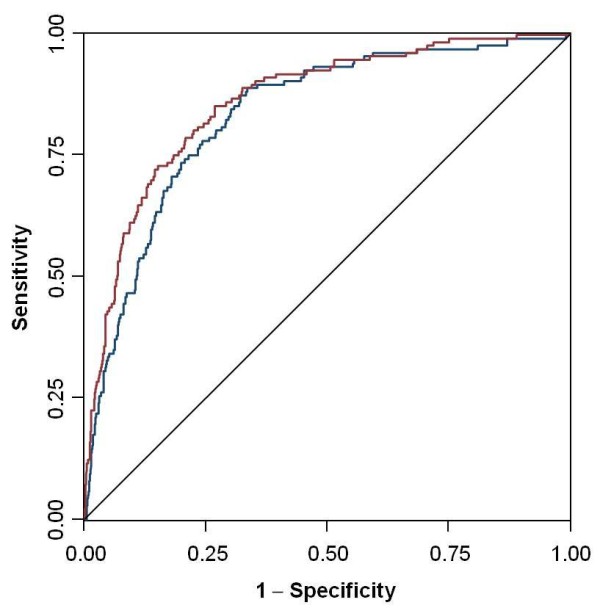
**Comparison of the areas under the receiver operating characteristics (AUC/ROC) between the final logistic regression models excluding and including the use of the pan-scan**.

It is noteworthy that the addition of the pan-scan to a model containing only the ISS, GCS, age and duration of emergency department care increased the area under the ROC curve from 0.837 to 0.864 (or 2.7%, Figure 2). However, the contribution of the pan-scan to the explained variance increased with higher injury severity (Table 5).

**Table 5:** Results from multivariable logistic regression analysis of mortality, stratified by ISS quartiles.

	Odds Ratio* (95% CI)		AUC/ROC†		
ISS quartile	Pan-scan available but not performed	Pan-scan performed	Limited model‡	Full model§	Additional variance explained by pan-scan use
First, 10 to 18	0.35 (0.09 to 1.46)	0.15 (0.03 to 0.71)	0.874	0.885	0.011
Second, 19 to 25	0.99 (0.33 to 2.95)	0.22 (0.08 to 0.61)	0.923	0.936	0.013
Third, 26 to 33	1.09 (0.12 to 10.14)	0.14 (0.03 to 0.64)	0.854	0.901	0.047
Fourth, 34 to 75	0.58 (0.13 to 2.55)	0.16 (0.08 to 0.36)	0.735	0.806	0.071

*Compared to the control period with conventional staged work-up (Odds Ratio 1.00), adjusted for age, ISS, GCS and emergency department time. †Area under the receiver operating characteristic curve. ‡Prognostic model containing only age, ISS, GCS and emergency department time. §Prognostic model containing only age, ISS, GCS, emergency department time and use of the pan-scan. GCS, Glasgow Coma Scale; ISS, Injury Severity Score.

## Discussion

In this study, the introduction of a liberal single-pass pan-scan policy was accompanied by a marked decline in mortality rates amongst patients with severe and multiple trauma.

A detailed analysis suggested that both the pan-scan itself and the general availability of a pan-scan contributed to the observed survival benefit. Thus, one might speculate of the pan-scan as a surrogate for enhanced process quality (including damage-control resuscitation and surgery) rather than an intervention with a causal impact on trauma outcomes. This was accompanied by significantly faster work-up times in the emergency department.

While sounding trivial, it cannot be stressed enough that the pan-scan is, no less, no more, a diagnostic tool that fits into a complex concept of early trauma care. As such, it must first shift the pre-test probability of injuries and influence clinical decision-making before it can affect therapeutic results.

This study did not investigate accuracy, diagnostic yield or the frequency of missed injuries with either work-up strategy. We also did not study the implication of pan-scan findings with regard to treatment plans. Within the hierarchy of diagnostic test research, our work addressed the efficiency of a modern imaging modality in terms of patient outcomes, presuming that it is adequately accurate and capable to influence management decisions. Yet, little is known about the distinct steps between diagnostic accuracy and patient outcomes.

In a prospective study that enrolled 284 patients with blunt trauma, emergency physicians considered 311 of 1102 (28.2%) scans of the head, neck, chest, abdomen and pelvis to be unnecessary [34]. Of those, 52 (16.7%) showed injuries, and, if omitted, would have led to false-negative results. However, only two injuries (i.e. a thoracic vertebral burst fracture with paralysis, and a subarachnoid bleeding) were considered of immediate therapeutic consequence (0.6%, 95% CI: 0.01 to 2.3%).

In a recent study from the UK, the trauma pan-scan detected 17 unanticipated injuries in 138 patients, posing immediate therapeutic consequences in three cases (two laparotomies and one chest tube) [35].

In another study of 329 patients with blunt trauma, 18 emergency physicians showed a high (69.9 to 100%) sensitivity in ruling out injuries without a pan-scan if they ranked the pre-test probability very low [36]. Sensitivity for different body regions like the head, cervical spine, chest, abdomen, pelvis, and the thoracolumbar spine, however, subsequently decreased with higher grades of pre-test probabilities of injuries.

Patients who actually underwent a pan-scan appeared to be more seriously injured than those who had a staged diagnostic work-up. There are two possible explanations for this imbalance that needed adjustment by multivariable analysis. On the one hand, patients with a high clinical pre-test probability of serious injuries (apart from the injury mechanism) may have been scheduled more frequently to a pan-scan (true-positive assumption). This interpretation is supported by the higher number of surgical procedures, a higher incidence of ARDS and MOF, and a longer duration of ICU stay in the pan-scan group.

On the other hand, the higher ISS may represent the typical stage migration phenomenon associated with the pan-scan, meaning that individual injuries are classified more severe because of their morphological appearance on CT images (false-positive assumption). For example, it has been shown that so-called occult pulmonary contusions (i.e. those only detectable on CT scans) increase the average ISS but do not lead to higher complication rates, mortality, or resource consumption compared to patients without lung contusions [37]. This assertion is supported by the larger proportion of chest injuries in the pan-scan group, specifically by the higher average AIS_Chest _in the pan-scan group.

A key finding of this study is that the attributable effect of the pan-scan to trauma survival is small compared to other major predictors like injury severity or the neurological status on admission. Apart from beneficial collateral effects of having a pan-scan available, patients who actually underwent a scan had a detectable extra reduction in overall mortality. The observed OR of 0.17 must, however, not be interpreted as a risk ratio (RR). The correct translation "patients who died were less likely to undergo a pan-scan" is entirely different to the common belief that "patients who underwent a pan-scan were less likely to die". The associated, unadjusted RR is 0.53 (95% CI 0.35 to 0.81), and the risk difference is 7%.

Based on the multivariable model presented here, the pan-scan explained 2.7% of the overall variance in mortality. One may consider this a remarkable effect size, given the 90.2% upper limit of variance in trauma mortality explained by the most sophisticated prognostic model currently available, the Revised Injury Severity Classification, or RISC score [38].

The contribution of the pan-scan to predicted mortality gradually increased with higher injury severity, meaning that more severely injured patients may earn greater benefits from a pan-scan than less severely injured patients.

This underlines the need for new triage criteria to avoid unnecessary convenience scans in less severely injured patients, and to restrict exposure to extra radiation to patients who are also most likely to achieve an extra benefit- in health-economic terms, to optimize the ratio of intangible costs to gains. Given the evidence available, the pan-scan is likely to be justified in those patients who deserve it (namely those with the highest trauma load) [32,39].

One possible explanation for the small overall effect size is that during the first months of the intervention period the pan-scan was interrupted if an intracranial hemorrhage was detected and continued after neurosurgical intervention. Since the introduction of the 64-slice scanner in April 2006 and accelerated examination times, the pan-scan is now completed regardless of cerebral bleedings as the delay is only minimal. Because of the limited sample size, the possible effect of this hardware and policy change was not investigated further.

A clear drawback is the retrospective and observational character of this study, and the limited sample size. Although a more detailed dataset than that of the federal register was available, and standard multivariable modeling was applied, the best-fitted model explained only 86% of variance in mortality. In addition to residual confounding caused by unmeasured demographic and injury variables, the dataset may have lacked important prognostic items of different phases of surgical stabilization and definitive care. We also did not distinguish between early and late deaths, or analyzed the underlying causes of mortality in particular. Future research may reveal whether the pan-scan has a significant role in reducing preventable deaths. Finally, we made no attempts to evaluate why surgeons opted against a pan-scan despite eligibility of the patient and availability of the technology.

Apart from all limits, the present data stress an important bias which should be taken into account in the health-technology assessment of the single-pass pan-scan for trauma. The still ongoing trend towards improved survival, probably caused by complex changes in the structural and process quality of trauma care, must be carefully considered before assigning any observed benefit to a single intervention, regardless of its presumed and obvious advantages.

## Conclusion

The pan-scan has already become an integral part of trauma work-up in many European countries and the US, although there is no formal proof of its accuracy and effectiveness. This is similar to many innovations in surgery (like laparoscopic cholecystectomy) that fall under Buxton's law- it is always too early to rigorously evaluate a new technology until it is suddenly too late [40,41]. In this study, we observed a significant reduction in the raw and injury-severity-adjusted mortality after severe blunt trauma with the introduction of a liberal single-pass pan-scan policy. This survival advantage applied to patients who actually did and did not undergo a pan-scan, and may have been confounded by a generally improved process quality care. Thus, causal links between improved patient outcomes and the broad implementation of a compelling imaging tool like whole-body computed tomography should be interpreted with caution. Our findings could assist in the trade-off between benefits and potential harms (mainly exposure to radiation) of the pan-scan for trauma, and underline the need for further studies on its accuracy, diagnostic yield, and related changes in management decisions.

## Abbreviations

AIS: Abbreviated Injury Scale; ARDS: respiratory distress syndrome; AUC: area under the curve; ED: emergency department; GCS: Glasgow Coma Scale; ICU: intensive care unit; ISS: Injury Severity Score; MOF: multiple organ failure; PRB: units of packed red blood; ROC: receiver operating characteristics.

## Competing interests

The authors declare that they have no competing interests.

## Authors' contributions

MH, AW and DS conceived the study and took responsibility for the design, implementation and reporting of the study. MH, AW, CH, CG and VB contributed to the conduct of the study including participant recruitment and follow-up, data collection and management. All authors had full access to all data and take responsibility for their integrity. DS did all statistical analyses, and drafted the manuscript. All other authors critically revised it for important intellectual content and approved the final version for publication.

All authors declare that they accept full responsibility for the conduct of the study and controlled the decision to publish. All authors read and approved the final manuscript
